# Antileishmanial Activity of Dimeric Flavonoids Isolated from *Arrabidaea brachypoda*

**DOI:** 10.3390/molecules24010001

**Published:** 2018-12-20

**Authors:** Vinícius P. C. Rocha, Cláudia Quintino da Rocha, Emerson Ferreira Queiroz, Laurence Marcourt, Wagner Vilegas, Gabriela B. Grimaldi, Pascal Furrer, Éric Allémann, Jean-Luc Wolfender, Milena B. P. Soares

**Affiliations:** 1 Laboratório de Engenharia Tecidual e Imunofarmacologia, Instituto Gonçalo Moniz, Fundação Oswaldo Cruz (Fiocruz), Avenida Waldemar Falcão, 121, Candeal–Salvador-BA 40296-710, Brazil; viny_rocha@hotmail.com (V.P.C.R.); gabrielagrimaldi16.1@bahiana.edu.br (G.B.G.); 2 Departamento de Química, Universidade Federal do Maranhão, São Luiz 65080-805, MA, Brazil; claudiarocha3@yahoo.com.br; 3 School of Pharmaceutical Sciences, EPGL, University of Geneva, University of Lausanne, CMU, 1, Rue Michel Servet, 1211 Geneva, Switzerland; emerson.ferreira@unige.ch (E.F.Q.); laurence.marcourt@unige.ch (L.M.); pascal.furrer@unige.ch (P.F.); eric.allemann@unige.ch (E.A.); jean-luc.wolfender@unige.ch (J.-L.W.); 4 UNESP-Campus Experimental do Litoral Paulista, Praça Infante Dom Henrique s/n°, Parque Bitaru, São Vicente–SP 11330-900, Brazil; vilegasw@clp.unesp.br

**Keywords:** *Leishmania*, flavonoids, high content, *Arrabidaea brachypoda*

## Abstract

Leishmaniasis are diseases caused by parasites belonging to *Leishmania* genus. The treatment with pentavalent antimonials present high toxicity. Secondary line drugs, such as amphotericin B and miltefosine also have a narrow therapeutic index. Therefore, there is an urgent need to develop new drugs to treat leishmaniasis. Here, we present the in vitro anti-leishmanial activity of unusual dimeric flavonoids purified from *Arrabidaea brachypoda*. Three compounds were tested against *Leishmana* sp. Compound 2 was the most active against promastigotes. Quantifying the in vitro infected macrophages revealed that compound 2 was also the most active against intracellular amastigotes of *L. amazonensis*, without displaying host cell toxicity. Drug combinations presented an additive effect, suggesting the absence of interaction between amphotericin B and compound 2. Amastigotes treated with compound 2 demonstrated alterations in the Golgi and accumulation of vesicles inside the flagellar pocket. Compound 2-treated amastigotes presented a high accumulation of cytoplasmic vesicles and a myelin-like structure. When administered in *L. amazonensis*-infected mice, neither the oral nor the topical treatments were effective against the parasite. Based on the high in vitro activity, dimeric flavonoids can be used as a lead structure for the development of new molecules that could be useful for structure-active studies against *Leishmania*.

## 1. Introduction

Leishmaniasis is a complex of diseases caused by protozoan parasites belonging to the genus *Leishmania* and transmitted by the bite of the female sand fly vector. The disease is endemic in 98 countries. Around 58,000 cases of visceral leishmaniasis and 220,000 cases of the cutaneous disease are officially reported per year worldwide [1]. The first-line treatment is performed with the pentavalent antimonials. Although still used, this chemotherapy presents several limitations, such as serious side effects, including patient death, prolonged course of treatment and emergence of drug resistance. Second-line drugs such as amphotericin B (AMB) and miltefosine can be used. However, similar to antimonials, these medicines are also associated with serious side effects, high cost and resistance [2]. Leishmaniasis represents a large social and economic burden. Due to the limitations of the current chemotherapy available, there is an urgent need to discover new drugs for the treatment of these devastating and neglected diseases.

*Arrabidaea brachypoda* (D.C.) is a native plant in Brazil, widely distributed throughout different biomes, belonging to the Bignoniaceae family, which encompasses 120 genera and approximately 800 species of plants, which constitute important components of neotropical forests. Phytochemical studies indicated that the *Arrabidaeae* genus is a source of *C*-glucosylxanthones, phenylpropanoids, flavonoids, anthocyanidins, allantoins and triterpenes [3,4]. In traditional medicine, species of this genus are used for different therapeutic purposes, such as astringent, anti-inflammatory, antimicrobial, antitumor and healing [5]. 

In Brazil, *Arrabidaea brachypoda* known as “cervejinha do campo”, is popularly used to treat kidney stones and arthritis, and has presented significant anti-inflammatory activity in experimental models [6,7]. Moreover, the anti-*Plasmodium falciparum* activity of other structurally related benzopyrano[4,3-b]benzopyran has been reported [8]. We have recently demonstrated the in vitro anti-*Trypanosoma cruzi* activity of the aqueous ethanol extract of the roots from *A. brachypoda* and its CH_2_Cl_2_ fraction. A follow-up investigation of the constituents from the CH_2_Cl_2_ fraction revealed the presence of three new dimeric flavonoids with different anti-*T. cruzi* activity in vitro and in an in vivo model of acute Chagas disease [7]. Based on the potential of the three dimeric flavonoids from *A. brachypoda* as anti-*T. cruzi* compounds, we investigated the anti-*Leishmania* activity of dimeric flavonoids **1**–**3** in vitro and their possible mechanism of action. Moreover, the most active flavonoid was selected for in vivo testing in a model of cutaneous leishmaniasis.

## 2. Results

### 2.1. In Vitro Activity Against Leishmania Promastigotes and Cytotoxicity to Mammalian Cells

**Figure 1:**
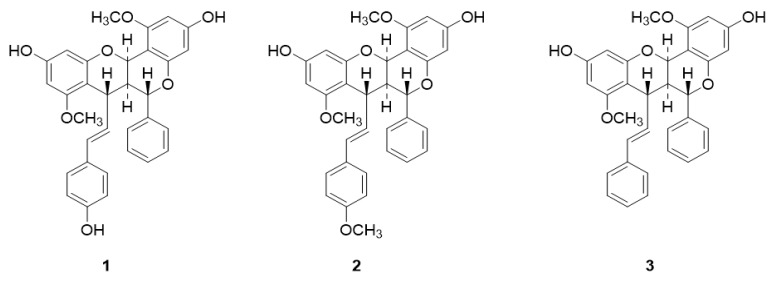
Structure of the tested compounds, Brachydin A (**1**), Brachydin B (**2**), and Brachydin C (**3**).

The purified compounds from *A. brachypoda* were tested against different species of *Leishmania*, namely *L. amazonensis*, *L. infantum* and *L. braziliensis*. First, *Leishmania* promastigotes were submitted to the treatment with compounds **1**–**3** (Figure 1) at concentrations ranging from 0.25 to 20 μM for 72 h. Compound **1** was not active against any *Leishmania* species at the tested concentrations, while compounds **2** and **3** presented similar potency and were active against all three *Leishmania* species. AMB, used as a positive control, showed a higher potency against promastigotes than the dimeric flavonoids assayed (Table 1).

**Figure 2:**
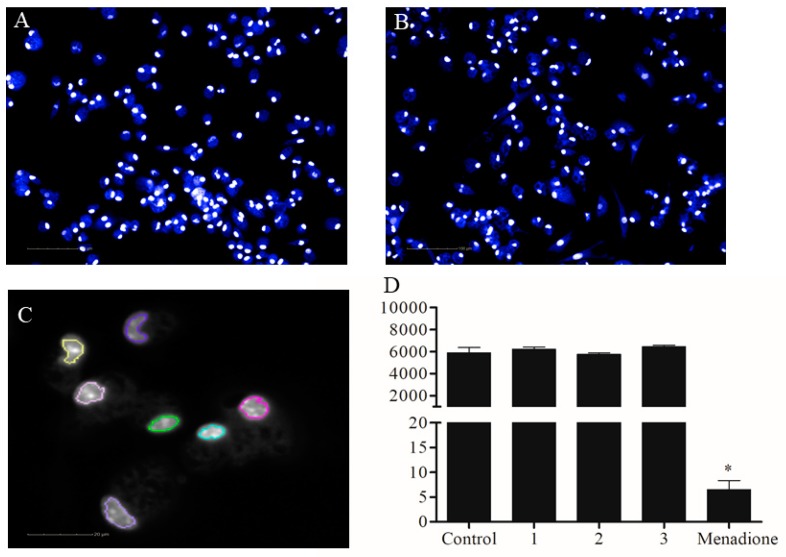
Automated drug toxicity assay. Macrophages from peritoneal exudate were collected from BALB/c mice. Cells were treated with compounds **1**–**3** at 50 µM or with menadione at 50 µM during 72 h. (**A**) Non-treated macrophages. (**B**) Macrophages treated with compound **2** at 50 µM during 72 h. (**C**) Nuclei regions selected based on the “find nuclei” building block algorithmic from Harmony software. (**D**) Graph showing the nuclei number, the parameter used to quantify the number of cells after the treatment. The graph shows one experiment of two performed in quadruplicate. Bars are mean ± standard deviation (SD). * *p* < 0.05, one-way ANOVA analysis of variance related to untreated control. Scale bar = 100 µm (**A**,**B**) and 20 µm (**C**).

Compounds **1**–**3** were first tested against non-infected primary macrophages to identify the toxic profile of the compounds in mammalian cells. The CC_50_ of compounds **1**–**3** based on Alamar Blue metabolism are shown in Table 1. All dimeric flavonoids tested did not present toxicity to peritoneal macrophages at 20 µM (Table 1). The high content analysis was applied to automatically quantify the nuclei number after 72 h of treatment. The automated quantification revealed no reduction in cell number after the treatment at 50 µM. On the other hand, menadione, a known cytotoxic compound [9], significantly reduced the number of macrophages at the same concentration (Figure 2). This result is in agreement with molecules containing the benzopyrano[4,3-b]benzopyran structures, as shown by previously published work [8].

### 2.2. Activity Against Amastigotes of L. amazonensis

**Figure 3:**
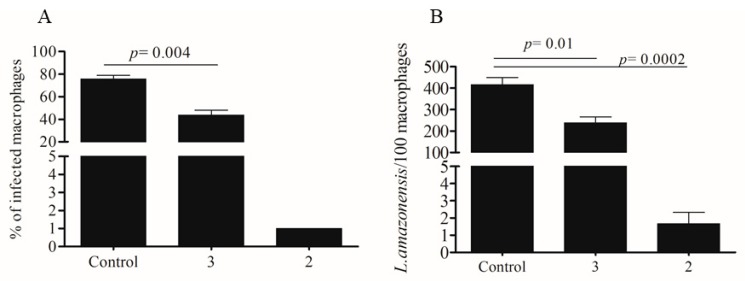
Effect of compounds **2** and **3** against amastigotes of *L. amazonensis*. Macrophages from peritoneal exudate were collected from BALB/c mice and infected with *L. amazonensis* promastigotes in the stationary growth phase during 6 h. The cells were washed and incubated during 24 h without treatment. After this period, the macrophages were treated with the compounds at 6 µM for 48 h. (**A**) Percentage of infected cells was evaluated by counting of 100 cells as well as the parasite number. (**B**). *p*-value was measured by unpaired t-test compared to control group.

Peritoneal exudate macrophages were infected with *L. amazonensis* and treated using non-toxic concentrations of dimeric flavonoids. Compound **1** was inactive against intracellular parasites, at 20 µM (Table 1). Compounds **2** and **3** were assayed at 6 µM and significantly reduced the percentage of infected peritoneal macrophages, as well as the number of amastigotes (Figure 3). It is noteworthy that compound **2** almost cleaned the infected cells at 6 µM (Figure 3). The IC_50_ concentrations against amastigotes are shown in Table 1. AMB was more active against intracellular amastigotes than the tested compounds. Based on the highest activity against intracellular parasites and the low toxicity to the host cell, compound **2** presented a higher selective index than compound **3**: 9.1 and 3.2, respectively (Table 1).

**Figure 4:**
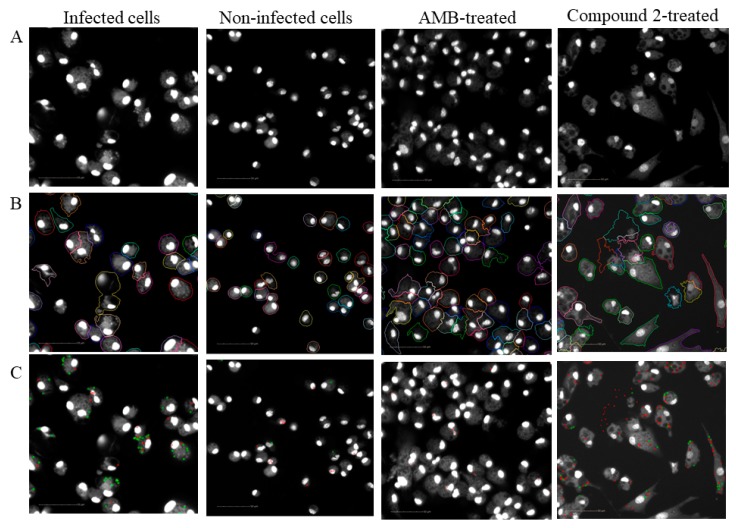
Image analysis process. Representative images of macrophages infected with *L. amazonensis*, non-infected control (mock), AMB-treated infected cell at 2 µM and compound 2-treated infected cell at 10 µM: (**A**) raw image acquired using the Hoechst channel; (**B**) cytoplasm segmentation based on nuclei selection using the “find cytoplasm” building block. Different colours are used to visually distinguish the cells; (**C**) discrimination between real parasites in the cytoplasm region (green) and non-parasite spot in the cytoplasm (red). Scale bar = 50 µm.

**Figure 5:**
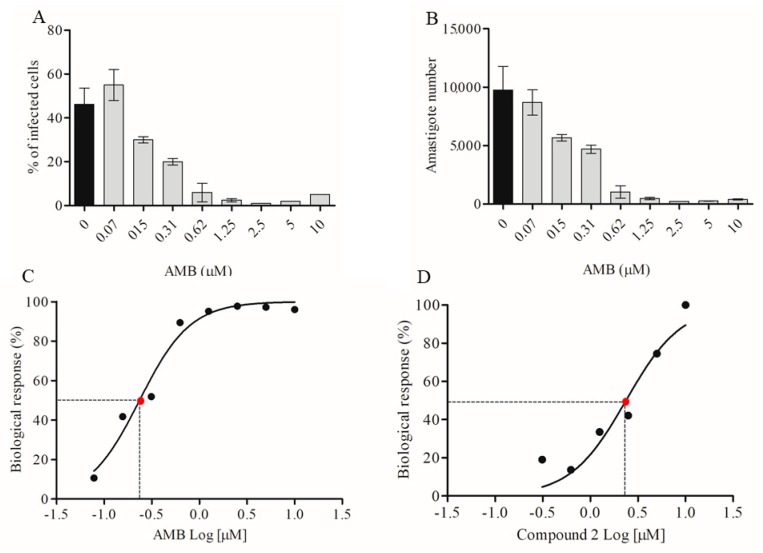
Automated quantification of amastigotes after treatment. (**A**,**B**) Infected cells were treated with AMB at different concentrations. The automated image segmentation and parasites quantification workflow were applied to determine the percentage of infection as well as the number of amastigotes. Bars represent the mean ± SD of one representative assay performed with eight replicates. (**C**,**D**) Quantification of the biological response after the treatment with AMB and compound **2** at concentration ranging from 0.15 to 10 µM. The correlation coefficient (r2) is 0.97 for AMB and 0.65 for compound **2**. Red dot means the logarithmic concentration related to 50% biologic response.

The automated image analysis was also applied to quantify the amastigote number after the treatment. After cell fixation and nuclei staining with Hoechst, the 96-well plates were submitted to image acquisition using the Operetta High-Content System. The cell segmentation was performed following the building blocks of image analysis created in the Hoechst 33342 reagent fluorescence channel (Figure 4). Intracellular parasites were selected in the cytoplasmic region using fluorescence intensity parameters, based on non-infected controls. AMB was used to validate the automated counting of amastigotes after treatment. Infected macrophages were treated with concentrations ranging from 0.07 µM to 10 µM during 72 h. This treatment presented a concentration-response relationship, regarding the percentage of infected cells as well as the total number of amastigotes in each well (Figure 5A,B). The IC_50_ calculated, based on the logarithmic concentration of AMB and the reduction of amastigotes number (% biological response) was 0.23 (Figure 5C), similar to previously published concentrations [10]. 

The same protocol for image analysis was applied to quantify the biological response of compound **2**, the most active dimeric flavonoid tested, as a single drug or in combination with AMB. The automated quantification of amastigotes presented an IC_50_ concentration equal to 2.4 ± 0.5 µM (Figure 5D). This value is similar to the IC_50_ concentration calculated by the manual counting of amastigotes (Table 1), validating again the automated method of parasite counting.

Combined treatment was performed using AMB and compound **2**. The sum of FIC generated a value of 1.5, corresponding to an additive effect relationship between these drugs. This value, slightly higher than the established cut-off value of 1, indicates that there are no interactions between them [11,12]. 

### 2.3. Electron Microscopy

**Figure 6:**
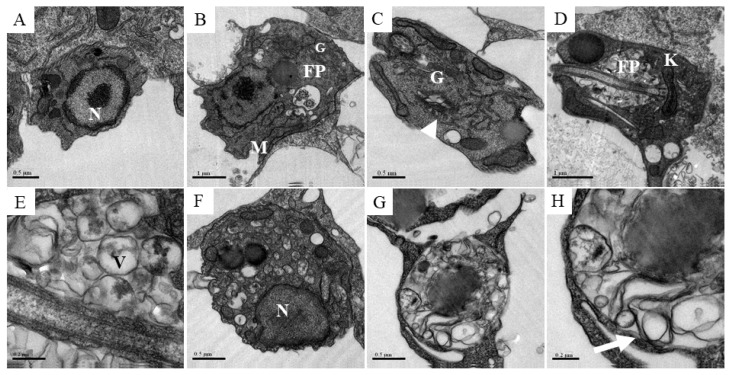
Ultrastructural analysis of amastigotes after the treatment with compound **2**. Peritoneal macrophages from BALB/c mice were infected with stationary phase *L. amazonensis* promastigotes and treated with compound **2** at 6 µM during 24 and 48 h. (**A**,**B**) Control amastigotes showing normal organelles, such as nuclei, mitochondria, flagellar pocket and Golgi. (**C**,**D**) Amastigotes treated with compound **2** during 24 h presenting the opening of Golgi (arrowhead) and vesicle accumulation inside the flagellar pocket, respectively. (**E**) Zoomed image of vesicles inside the flagellar pocket highlighting the double and multiple membrane formation. (**F**,**G**) Increased accumulation of cytoplasmic vesicles in amastigotes treated with compound **2** during 48 h. (**H**) Zoomed image showing a myelin-like figure in the cytoplasm of an amastigote (arrow). N, nuclei; M, mitochondria; FP, flagellar pocket; G, golgi; K, kinetoplast; V, vacuole.

Transmission electron microscopy was performed in infected and treated macrophages to investigate the alterations induced by compound **2** in the intracellular amastigotes at the ultrastructural level. This assay was performed at low concentration, 6 µM, as well as reduced treatment time (24 and 48 h). As shown above, even high concentrations such as 20 µM and 50 µM were not able to generate toxicity to the macrophages. Non-treated cells presented amastigotes with rounded cell shape and normal organelle morphology, such as nuclei, mitochondria, flagellar pocket and Golgi apparatus (Figure 6A,B). Treatment with compound **2** at 6 µM for 24 or 48 h induced phenotypic alterations, such as enlargement of Golgi cisternae after 24 h (Figure 6C). In association with Golgi damage, the intracellular parasites presented accumulation of vesicles, characterized by a double membrane, inside the flagellar pocket (Figure 6D,E). Following treatment with compound **2**, the parasites accumulated multivesicular bodies (Figure 6F). These alterations led to an increase of intracellular vesicles and cytoplasmic disorganization, resulting in cell death (Figure 6G). Amastigotes carrying myelin-like figures were seen after treatment with compound **2** for 48 h (Figure 6H).

### 2.4. In Vivo Infection

**Figure 7:**
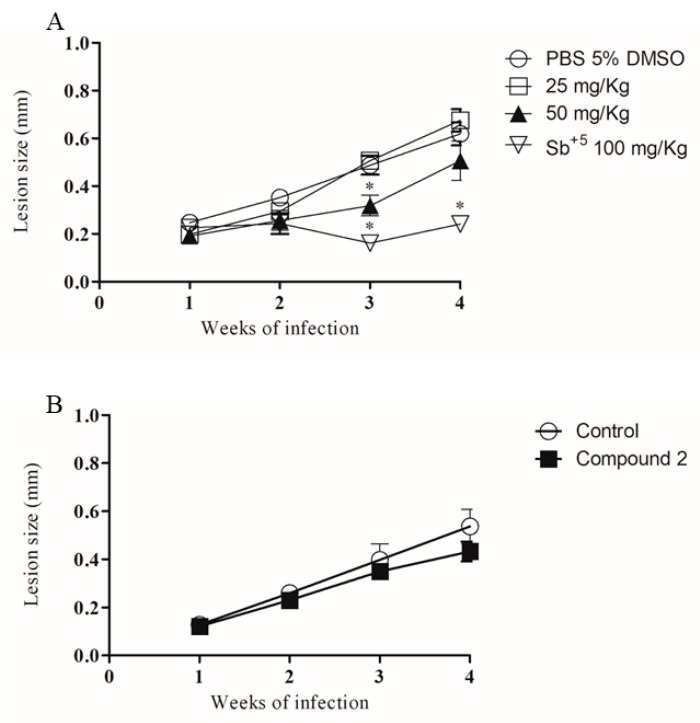
Pharmacological effect of compound **2** in an experimental model of cutaneous leishmaniasis. BALB/c mice were infected with 10^6^ *L. amazonensis* promastigotes in the stationary growth phase and lesion development was monitored for four weeks. Lesion size is expressed as mean ± SD of eight animals per group during the treatment by oral (**A**) or topical (**B**) route. * *p* < 0.05 (two-way ANOVA, followed by Bonferroni’s multiple comparison test).

Genetically susceptible BALB/c mice were infected in the ear dermis with *L. amazonensis* and treated by oral (25 and 50 mg/kg) or topical (1%) routes with compound **2** starting from the second week of infection. While the oral treatment with 25 mg/kg did not alter the lesion growth, treatment with 50 mg/kg of compound **2** significantly reduced the ear thickness in the second week of treatment (third week of infection). One week later, however, there was no statistically significant difference between the thickness of controls. Pentavalent antimonial (Glucantime^®^), used as positive control, significantly reduced the lesion size (Figure 7A). Daily treatment with topical formulation, however, did not reduce the lesion size compared to vehicle-treated controls (Figure 7B).

## 3. Discussion

The hydroalcoholic root extract from *A. brachypoda* presented pharmacological activity in vitro against trypomastigotes of *T. cruzi*. Anti-parasitic activity was found in the dichloromethane fraction. Next, a follow up investigation revealed the presence of unusual dimeric flavonoids, from which two molecules showed biological activity against *T. cruzi* in vitro and in vivo [7]. Based on these findings, the dimeric flavonoids were tested against *Leishmania*.

Our results showed a higher activity of compound **2** against intracellular parasites. Amastigotes are the parasitic life cycle form found in mammalian hosts, responsible for the infection maintenance in the organism [13]. It is known that promastigotes differ from amastigotes not only in terms of morphology but also with respect to their metabolism. Different biochemical pathways are up or down regulated according to the life cycle parasitic stage. This distinct biology reflects different developmental programs to adapt *Leishmania* for intra- or extracellular survival inside the mammalian or vector host, respectively [14,15,16]. Therefore, the stage specific biology may influence the parasite susceptibility to chemicals [17,18]. Moreover, the presence of a host cell may also generate different results when compared to an assay only involving promastigotes. Compound and host features, such as permeability to the cell and parasitophorous membrane or cell metabolism, are key parameters able to give a drug more potency against intracellular amastigotes [18]. In this work, compound **2** (brachydin **2**) has been shown to be more active, possibly, because the presence of the methoxyl group is necessary to improve membrane penetration. The substituents on the C ring probably play a key role in the increase in lipophilicity and consequently the penetration of the compound through the host and protozoan cell membranes, since the brachydines differ only in the substituent group. The capacity of the *Leishmania* to evade or resist the innate immunological response is pivotal to the maintenance of infection. Therefore, the ability of the drug to rescue the macrophage antimicrobial response can also be more potent against infected cells [19,20].

In this work, the combination of compound **2** with AMB was assayed. Based on the sum of FIC, compound **2** does not interact with AMB to improve the efficacy against intracellular parasites (synergic effect). Drug combination is an important strategy in the development of new treatments, particularly in infectious diseases. This approach can reduce the dose, time and the cost of a such pharmacological treatment, in addition to overcome the weak activity of the individual drug. Moreover, by acting through different pharmacodynamics, the combination therapy can improve treatment, as well as reduce the development of drug resistance [21]. 

The ultrastructural analysis of compound **2** treated amastigotes showed the ability of the compound to induce cell lesion which progressed to parasitic death. The Golgi damage associated with accumulation of vesicle inside the flagellar pocket suggests the impairment of endocytic pathways in amastigotes. Vesicle accumulation may reflect the defect in the release process or increased production and exocytosis. Vesicle traffic inside amastigotes is important for nutrient acquisition, intracellular communication, virulence and host/pathogen interaction [22,23].

Interestingly treatment with compound **2** reduced the parasitemia of *T. cruzi* Y strain-infected mice [7]. In that case, treatment was performed during five days at 100 mg/kg by oral route. This promising result suggests that compound **2** is absorbed by oral route and kills parasites in the blood. In contrast to the acute phase of experimental Chagas disease, during *Leishmania* infection, the drug needs to be distributed into internal organs, such as draining lymph nodes, spleen and liver, and towards the site of infection to exert its pharmacological activity [24,25]. Conceivably, the result found here may reflect the incapability of compound **2** to achieve effective concentration in the infected ear by oral route. The treatment of *Leishmania*-infected mice may need to be longer than three to four weeks. To avoid in vivo toxicity, the highest daily dose was reduced to 50 mg/kg. The ineffectiveness of the topical treatment may also reflect the pharmacokinetics problems, in this case related to drug absorption by the skin.

The promising in vitro results of compound **2** were not reproduced during the in vivo experiments, by factors related to the drug/formulation pharmacokinetics, as discussed above, or even host-related mechanisms, such as liver metabolism. However, this work presented a novel compound with high in vitro activity against amastigotes that can be used for lead optimization and structural-activity relationship studies [26]. Our ultimate aim is to generate a highly selective drug, based on compound 2 and improve pharmacokinetic features, following high content drug screening and in vivo assays.

## 4. Materials and Methods 

### 4.1. Plant Material

*A. brachypoda* roots were collected in April 2010 at Sant’Ana da Serra farm João Pinheiro, Minas Gerais, Brazil. The plant was identified at the ICEB of the José Badine Herbarium of the Federal University of Ouro Preto by Prof. Maria Cristina Teixeira Braga Messias. A voucher specimen (no. 17935) has been deposited at the Herbarium of the Federal University of Ouro Preto, Brazil. The plant was collected in accordance with Brazilian authorities (SISGEN N°n° A451DE4).

### 4.2. Extraction and Isolation of Compounds Brachydin A *(**1**)*, Brachydin B *(**2**)*, and Brachydin C *(**3**)*

The dimeric flavonoids **1**–**3** have been obtained using the same process previously described [7]. NMR and HRMS have determined the structures of the compounds. The purity of each compound was determined by UHPLC-HRMS analysis. For all compounds the purity was above 98%.

### 4.3. Mice

Female, 4 to 8-weeks-old C57BL/6 or BALB/c mice were obtained from the Animal Facilities of the Gonçalo Moniz Institute-FIOCRUZ (Salvador, Brazil). Animals were housed in temperature-controlled rooms (22–25 °C) under a 12:12 h light-dark cycle and provided with rodent diet and water *ad libitum*. Animals were handled according to the NIH guidelines for animal experimentation. All procedures described here had prior approval from the local animal ethics committee (approval number 018/2015).

### 4.4. Parasites

*L. amazonensis* (MHOM/BR88/BA-125) were routinely passed in C57BL/6 mice to maintain virulence. Parasites were isolated from the popliteal lymph node after cultivation in biphasic Novy–Nicolle–MacNeal (NNN) medium containing Schneider’s insect medium (Sigma Aldrich, St. Louis, MO, USA) supplemented with 10% fetal bovine serum (Gibco Laboratories, Waltham, MA, USA) and 50 µg/mL of gentamicin (Hipolabor, Belo Horizonte, Minas Gerais, Brazil) at 24 °C. The obtained promastigotes were transferred to complete Schneider’s insect medium, cultivated until stationary growth and used in the following experiments. *L. braziliensis* (MHOM/BR88/BA-3456) and *L. infantum* (MHOM/BR2000/Merivaldo2) were cultivated until reaching the stationary phase in Schneider’s insect medium supplemented with 10% or 20% fetal bovine serum, respectively. The virulence of both species was maintained by passages in BALB/c female mice.

### 4.5. Activity of Dimeric Flavonoids Against Axenic Promastigotes of Leishmania

Stationary phase *L. amazonensis*, *L. braziliensis* and *L. infantum* promastigotes were seeded in a 96-well plate at a density of 2 × 10^6^ parasites/mL in 200 μL of complete Schneider’s insect medium. Cells were treated with dimeric flavonoids at concentrations ranging from 0.25 to 20 µM for 72 h. AMB was used as positive control (0.04 to 3 µM). Promastigotes viability was measured by Alamar Blue (Invitrogen, Carlsbad, CA, USA) metabolism and colorimetric readings were performed at 570 and 600 nm. The blank used in this assay was the medium plus Alamar Blue without parasites. The IC_50_ concentration was calculated based on the percent inhibition of parasite growth, related to negative controls, and accessed through concentration logarithm values followed by nonlinear regression curve fit. Analyses were performed using the GraphPad Prism version 5.01 (GraphPad Prism, San Diego, CA, USA).

### 4.6. Cytotoxicity Assay

Peritoneal exudate macrophages from BALB/c mice were isolated as previously described, after five days of thioglycolate (Sigma Aldrich) stimulation [27]. The cells (5 × 10^4^ per well in 200 mL) were cultured in 96-well plates in the presence of compounds **1**–**3** at concentrations ranging from 2.5 to 20 µM during 48 h at 37 °C in 5% CO_2_. Cells were incubated with 20 µL of Alamar Blue per well (Invitrogen) for an additional 24 h. Colorimetric absorbance readings were performed at 570 and 600 nm and used to calculate the percentage of growth inhibition after the treatment. The blank used in this assay was the medium plus Alamar Blue without the cells. The cytotoxic concentration to 50% of macrophages (CC_50_) was calculated through non-linear regression using GraphPad Prism version 5.01.

### 4.7. In Vitro Macrophage Infection with L. amazonensis

Peritoneal exudate macrophages from BALB/c mice were obtained after five days of thioglycolate stimulation (Sigma Aldrich) and infected with *L. amazonensis* [27]. Cells (2 × 10^5^/mL) were plated in 24-well plates containing 13 mm-diameter glass coverslips in Dulbecco's Modified Eagle Medium (DMEM) containing 10% fetal bovine serum (Gibco Laboratories) and incubated overnight at 37 °C with 5% CO_2_. Macrophages were infected with stationary phase promastigotes at a ratio of 5 parasites per host cell for 6 h at 35 °C, 5% CO_2_. Each well was washed to remove free parasites, and cultures were incubated for 24 h under the same conditions to allow promastigote to amastigote differentiation. Infected cells were incubated with several concentrations of dimeric flavonoids (0.24 to 20 μM) or AMB (10 to 0.07 µM) during 72 h at 37 °C, 5% CO_2_. The cells were fixed with ethanol and stained with conventional haematoxylin and eosin (H&E). The inhibitory concentration for 50% of parasites (IC_50_) was calculated based on the percent reduction of amastigote number compared to negative control.

### 4.8. Image Acquisition 

The infected macrophages were fixed with a paraformaldehyde 4% in phosphate buffered saline during 20 min at room temperature, after 72 h of treatment. Cells were stained with Hoechst 33342 (Thermo Fischer Scientific, Waltham, MA, USA) at 16 µM. Images were acquired using the Operetta High-Content System in non-confocal mode and 20X air objective, for further segmentation and quantification (Perkin Elmer, Waltham, MA, USA). Pictures from five fields per well were analysed for reliable statistics.

### 4.9. Automated Image Analysis 

The images of infected cells were analyzed using the Harmony software version 3.5.2 (Perkin Elmer) using an algorithm provided by the software building blocks. Nuclei were detected using the Hoechst fluorescence channel, as well as the cytoplasm. The intracellular amastigotes were detected as spots in the cytoplasmic selected region. A mock control was used to remove the background spot detection and select the correct spots as amastigotes. Fluorescence intensity parameters, such as median, mean, maximum intensity and contrast, were applied to automatically select the amastigotes in infected cells. Nuclei detection allowed the quantification of cell number for quality control and cytotoxic evaluation while amastigote detection allowed the quantification of total number of parasites.

### 4.10. Calculation of Biological Response

The biological response of the tested compounds was determined as following: % of biological response = (X1 − Xx/X1 − X2) × 100, where X1 is the lowest activity (untreated cells, maximum of parasite number), X2 is the highest activity (non-infected cells) and Xx is the tested drug at different concentrations [28]. The percentage of biological response was determined based on the total number of amastigotes and indicates the percentage of parasite number reduction after the treatment, related to non-treated cell and corrected by the non-infected group. The IC_50_ was calculated using non-linear regression. Cytotoxicity was calculated based on the number of nuclei after the treatment. The percentage of cell decrease was calculated based on the untreated control. Calculations were performed using GraphPad Prism version 5.01.

### 4.11. Drug Combination

Combined treatment was performed with AMB at a maximum concentration of 2 µM. The fixed ratio combination was equal to 5. The combination was submitted to six serial dilutions. The percentage of biological response of combined drugs was used to calculate the fraction of inhibitory concentration (FIC). The combined effect was classified based on the combination index (CI), which is the sum of FIC generated: CI ≤ 0.5 indicates synergism (drug combination can exert biological response which are more than the sum of the effect of the drugs alone); 0.5 ≤ CI ≤ 4 indicates additivity (no interaction) and CI ≥ 2 indicates antagonism (drug combination can exert biological response which are less than the sum of the effect of the drugs alone) [12].

### 4.12. Transmission Electron Microscopy

Peritoneal exudate macrophages obtained from BALB/c mice were plated at a density of 10^6^ cell per well in 6-well plate. Adhered macrophages were infected with *L. amazonensis*, as previously described. Compounds were added at 6 µM, and cultures were incubated for 24 or 48 h at 35 °C 5% CO_2_. Infected and treated macrophage cultures were fixed in a solution of 2.5% glutaraldehyde, 2% formaldehyde and 2.5 mM CaCl_2_ in 0.1 M sodium cacodylate buffer pH 7.2, followed by post-fixation in 1% osmium tetroxide and 0.8% potassium ferricyanide in the same buffer. The macrophages were removed from the 6-well plate using cell scraper and the samples were submitted to acetone gradient dehydration. Finally, the material was embedded in Poly/Bed resin. Ultrathin sections were stained with uranyl acetate and lead citrate and observed under a JEM 1320 transmission electron microscope (JEOL, Tokyo, Japan).

### 4.13. In Vivo Infection with L. amazonensis

Female BALB/c mice, four to six-weeks-old, were infected in the right ear dermis with 10^6^ *L. amazonensis* promastigotes in stationary growth phase in 10 µL of saline. After one week of infection, mice were treated daily by oral or topical routes during the following three weeks. The ear swelling was monitored weekly, using a digital caliper (Mitutoyo, Kanagawa, Japan) and was determined as the difference in thickness between the infected and contra-lateral uninfected ear. The control groups received saline by oral route or the water emulsifying ointment by topical route. This later formulation was composed of emulsifying cetostearyl alcohol (type A) 30% *w*/*w*, liquid paraffin 20% *w*/*w* and white soft paraffin 50% *w*/*w*. The active compound was incorporated in the ointment as a 1% DMSO solution.

## Figures and Tables

**Table 1:** In vitro pharmacological activity of dimeric flavonoids.

IC_50_ (µM) Promastigotes *Leishmania* sp.					IC_50_ Amastigotes	
Compound	*L. amazonensis*	*L. braziliensis*	*L. infantum*	CC_50_ MΦ	IC_50_ *L. amazonensis*	SI
(**1**)	>20	>20	>20	>20	>20	NC
(**2**)	9.16 ± 1	7.05 ± 1	12.90 ± 3	>20	2.20 ± 0.09	9.1
(**3**)	10 ± 0.80	8.82 ± 3	18.36 ± 2	>20	6.25 ± 1.28	3.2
AMB	0.14 ± 0.01	0.11	0.05	>20	0.10 ± 0.02	200

AMB: amphotericin B; MΦ: macrophages; CC_50_: cytotoxic concentration to 50% of macrophages; IC_50_: cytotoxic concentration to 50% of parasites; SI: selective index (CC_50_/IC_50_).
